# Intravenous Idursulfase for the Treatment of Mucopolysaccharidosis Type II: A Systematic Literature Review

**DOI:** 10.3390/ijms25168573

**Published:** 2024-08-06

**Authors:** Walla Al-Hertani, Ravi R. Pathak, Obaro Evuarherhe, Gemma Carter, Carolyn R. Schaeffer-Koziol, David A. H. Whiteman, Ekaterina Wright

**Affiliations:** 1 Division of Genetics and Genomics, Boston Children’s Hospital, Harvard Medical School, Boston, MA 02115, USA; 2 Takeda Pharmaceuticals USA, Inc., Lexington, MA 02421, USA; ravi.pathak@takeda.com (R.R.P.); carolyn.schaeffer-koziol@takeda.com (C.R.S.-K.); 3 Oxford PharmaGenesis, Oxford OX13 5QJ, UK; obaro.evuarherhe@pharmagenesis.com (O.E.); gemma.carter@pharmagenesis.com (G.C.); 4 Takeda Development Center Americas, Inc., Lexington, MA 02421, USA; dahw.md@gmail.com (D.A.H.W.); ekaterina.wright@takeda.com (E.W.)

**Keywords:** enzyme replacement therapy, idursulfase, lysosomal storage diseases, mucopolysaccharidosis II, systematic literature review

## Abstract

Mucopolysaccharidosis type II (MPS II; Hunter syndrome) is a rare, X-linked disorder caused by deficient activity of the enzyme iduronate-2-sulfatase. Signs and symptoms typically emerge at 1.5–4 years of age and may include cognitive impairment, depending on whether patients have the neuronopathic or non-neuronopathic form of the disease. Treatment is available in the form of enzyme replacement therapy (ERT) with recombinant iduronate-2-sulfatase (idursulfase). A systematic literature review was conducted to assess the evidence regarding efficacy, effectiveness, and safety of ERT with intravenous idursulfase for MPS II. Electronic databases were searched in January 2023, and 33 eligible articles were found. These were analyzed to evaluate the effects of intravenous idursulfase and the overall benefits and disadvantages in patient subgroups. Studies showed that intravenous idursulfase treatment resulted in improved short- and long-term clinical and patient-centered outcomes, accompanied by a favorable safety profile. Patients with non-neuronopathic MPS II had more pronounced improvements in clinical outcomes than those with neuronopathic MPS II. In addition, the review identified that improvements in clinical outcomes are particularly apparent if intravenous idursulfase is started early in life, strengthening previous recommendations for early ERT initiation to maximally benefit patients. This review provides a comprehensive summary of our current knowledge on the efficacy of ERT in different populations of patients with MPS II and will help to inform the overall management of the disease in an evolving treatment landscape.

## 1. Introduction

Mucopolysaccharidosis type II (MPS II, Hunter syndrome, OMIM 309900) is a rare, X-linked disorder characterized by a deficiency of iduronate-2-sulfatase (I2S). Deficiency in I2S activity leads to accumulation of glycosaminoglycans (GAGs) within the lysosomes, resulting in multisystemic cell and organ dysfunction, significant morbidity, and a shortened life expectancy [1]. With an estimated incidence between 1 in 100,000 and 1 in 170,000 births, MPS II affects males almost exclusively, although a few symptomatic female patients have been identified [2,3]. MPS II is caused by pathogenic variants of the I2S gene (*IDS*), with phenotype severity dependent on the type of variant [1,3]. MPS II is characterized by marked genotypic and phenotypic heterogeneity, representing a continuum of disease severity. Based on a traditional clinical phenotype categorization, patients are typically classified as having either the non-neuronopathic (attenuated) form, which has no central nervous system (CNS) involvement, or the neuronopathic (severe) form, which has a broad spectrum of neurological involvement [1,3]. Age at onset and disease progression are heterogeneous; patients typically have a normal appearance at birth, with the initial signs and symptoms emerging between the ages of 18 months and 4 years, depending on disease severity [4]. Typical somatic signs and symptoms of MPS II include a large head (macrocephaly), thickened tongue (macroglossia), hoarse voice, joint stiffness, upper respiratory and ear infections, cardiovascular and respiratory abnormalities, umbilical/inguinal hernias, and organomegaly of the liver and spleen; accumulation of urinary GAGs (uGAGs) is also observed upon laboratory testing [4]. Approximately 60% of patients have neuronopathic MPS II and are likely to experience neuronopathic symptoms at about 3–4 years of age, including behavioral changes, attention difficulties, speech delay, cognitive decline, and poor performance at school [5].

Although there is no cure for MPS II, enzyme replacement therapy (ERT), particularly when initiated early, can help to manage symptoms and improve quality of life [4,6]. Intravenous (IV) idursulfase (Elaprase^®^) is an ERT that was approved in the USA in 2006 and in Europe in 2007 and is indicated for long-term treatment of MPS II [7,8]. This therapy, delivered on a weekly basis, replaces the deficient or absent I2S enzyme with a functional recombinant human version of I2S to restore enzyme activity and reduce GAG accumulation in affected tissues [9]. IV idursulfase is the only ERT approved for treatment of MPS II by the US Food and Drug Administration and the European Medicines Agency [10]. ERT stabilizes or improves many somatic disease manifestations of MPS II [11,12,13], but it has not been shown to cross the blood–brain barrier (BBB) in sufficient concentrations to address neuronopathic symptoms [3,6]. Several therapeutic agents are currently being investigated for neuronopathic MPS II in active clinical trials, which use novel fusion proteins to drive a peripherally delivered enzyme directly across the BBB [10]. Gene therapy is under development and may also have the potential to address both somatic and neuronopathic manifestations of MPS II [14].

The importance for early diagnosis to facilitate optimal treatment outcomes has been thoroughly discussed in MPS II and other lysosomal storage diseases, because progressive diseases may result in irreversible organ damage or dysfunction, which could be prevented with early treatment [2,3,15]. In 2022, MPS II was added to the US Recommended Uniform Screening Panel [16]; therefore, MPS II is expected to be incorporated into newborn screening procedures to help to drive early disease recognition and management across the USA.

The purpose of conducting this systematic literature review (SLR) is to assess the evidence regarding the efficacy, effectiveness, and safety of ERT with IV idursulfase for MPS II. We describe short- and long-term outcomes with IV idursulfase and also consider the overall benefits and disadvantages in patient subgroups, including those younger than 1.5 years, patients with neuronopathic MPS II, and patients with presymptomatic MPS II.

## 2. Methods

### 2.1. Registration and Methodology

The SLR protocol was registered with the international prospective register of systematic reviews PROSPERO (CRD42023434690) [17]. The review was conducted in accordance with the 2020 Preferred Reporting Items for Systematic Reviews and Meta-Analyses (PRISMA) guidelines [18]. 

### 2.2. Search Strategy

Publications were identified by systematic electronic searches of Embase (1974–present), MEDLINE (In-Process & Other Non-Indexed Citations and Ovid MEDLINE [1946–present]), the Cochrane Library (which comprises the American College of Physicians Journal Club, the Cochrane Database of Systematic Reviews, and the Cochrane Central Register of Controlled Trials), the Evidence-Based Medicine Reviews (EBMR) Database of Abstracts of Reviews of Effects, the UK National Health Service Economic Evaluation Database, the Health Technology Assessment Database, and all EBMR content. Searches were supplemented from 2021 onward with manual searches of relevant congress proceedings, including those from the Lysosomal Diseases Gordon Research Conferences, the Society for the Study of Inborn Errors of Metabolism annual symposia, the Lysosomal Disease Network Annual WORLD*Symposia*, the International Congresses of Inborn Errors of Metabolism, and the International Society for Pharmacoeconomics and Outcomes Research annual conferences. 

Reference lists of systematic reviews were cross-checked for any relevant sources. Search strings used to identify evidence are listed in Table S1. The search strings include a mixture of free text and Medical Subject Headings terms, combining terms for MPS II with terms for interventions. The searches were restricted to English language publications but were not limited by year or region. Electronic searches were run on 24 January 2023, and downloaded into EndNote (Clarivate, Philadelphia, PA, USA); duplicate entries were removed before eligibility screening.

### 2.3. Publication Eligibility

The titles and abstracts of all identified publications in the electronic searches were screened double-blind by two independent reviewers against the predefined eligibility criteria outlined in Table 1 to ensure all relevant evidence was captured and to minimize bias. Both reviewers determined whether publications met the eligibility criteria, and then resolved uncertainties or discrepancies by discussion or by escalation to a third reviewer. All irrelevant titles and/or abstracts were excluded, and reasons for exclusion were captured in accordance with PRISMA guidelines. Meta-analyses and clinical and observational studies were considered. Case studies published from 2017 onward were flagged during citation screening to assess their impact on scope. Case reports published before 2017 were excluded. Full-text review was conducted following initial screening to ensure article eligibility. 

### 2.4. Data Extraction and Outcomes

The data extraction process was performed by a single reviewer and independently checked for errors by a second reviewer. Any discrepancies were resolved through discussion or through the intervention of a third reviewer. For each included study, a risk of bias assessment was performed using the Risk of Bias 2 tool for randomized controlled trials (RCTs), the Risk Of Bias In Non-randomized Studies – of Interventions tool for nonrandomized studies, or the Joanna Briggs Institute critical appraisal checklist for systematic reviews and research synthesis (for meta-analyses). 

The SLR was performed with the aim of addressing the following questions in relation to the efficacy, effectiveness, and safety of IV idursulfase.

- How has IV idursulfase led to improved clinical or patient-centered outcomes in children and adults with diagnosed MPS II?
- What are the limitations of using the 6-min walk test (6MWT) as an isolated clinical indicator?
- What are the short- and long-term benefits of the use of IV idursulfase?
- Are the benefits of treatment with IV idursulfase based on presymptomatic diagnosis, phenotype, or age?
- What are the benefits, if any, of IV idursulfase treatment for patients with neuronopathic MPS II?
- Are there any benefits or disadvantages associated with the use of IV idursulfase in patients aged younger than 18 months or younger than 5 years?
- What are the outcomes with different doses of IV idursulfase?

To explore these questions, the following were collected as part of the data extraction process: study details and data regarding patient population (neuronopathic, non-neuronopathic, genotype, and gender), treatment (regimen, dosage, and discontinuation), baseline characteristics (age at diagnosis, symptom onset and treatment start, age group, race and ethnicity, and duration of treatment), safety and efficacy outcomes (GAG, cardiac, pulmonary, 6MWT, liver and spleen size, patient-reported, cognitive, growth-related, joint function, immunogenicity, and survival outcomes). To evaluate the impact of ERT on outcomes by age, studies were also grouped as follows: any infants (<18 months, yes or no), any preschool children (18 months–4 years, yes or no), any schoolchildren/adolescents (5–17 years, yes or no), and any adults (≥18 years, yes or no).

## 3. Results

### 3.1. Study Characteristics

**Figure 1:**
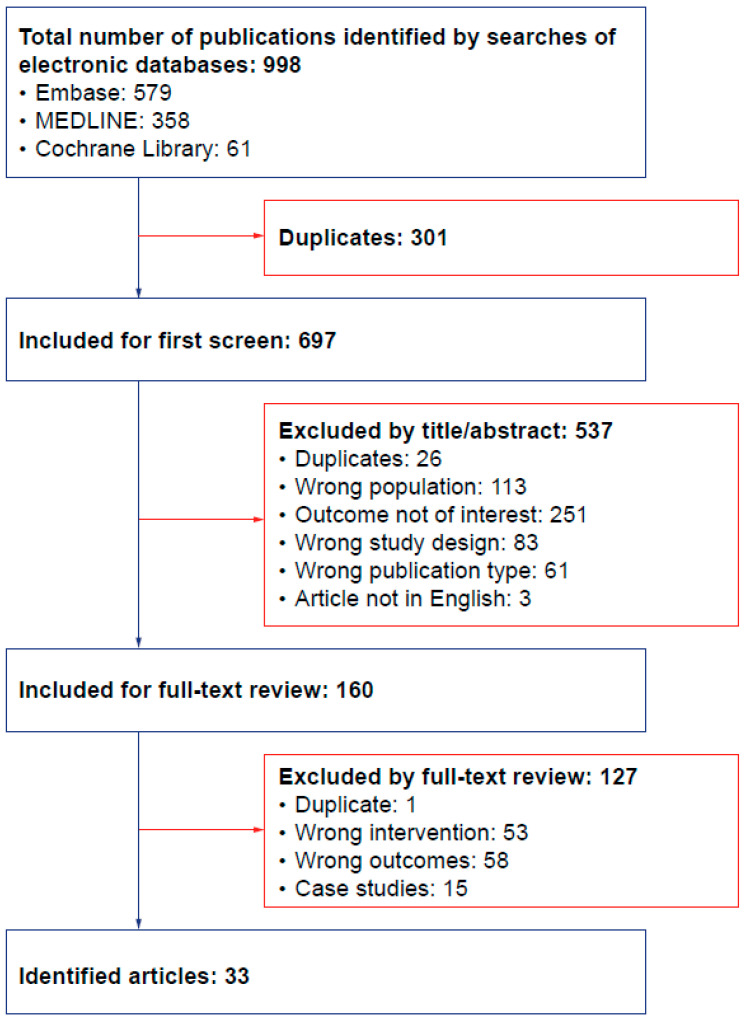
PRISMA diagram of studies included and excluded from the SLR analysis. PRISMA—Preferred Reporting Items for Systematic Reviews and Meta-Analyses; SLR—systematic literature review.

The screening process used for identification and shortlisting of publications is summarized in the PRISMA flow chart (Figure 1). Overall, 33 articles were identified from the searches (Table 2). Among these, 26 described observational studies, 3 reported randomized clinical trials (1 also including findings from an open-label extension), and 4 showed results only from open-label studies. Of the three articles describing RCTs, two illustrated the pivotal phase 2/3 RCT, including the primary results [19] and a post hoc immunology analysis (from the randomized and open-label extension periods) [20], and one described a phase 1/2 RCT [13]. Of the four articles relating only to open-label studies, two described a pediatric, phase 4, single-arm, open-label study (one article reported the main results [12] and the other discussed the post hoc immunology analysis [21]); one reported results from the long-term extension period of the pivotal phase 2/3 clinical trial [11]; and one described a single-arm, open-label study in Japanese adults [22].

Of the 26 identified observational studies, there were 11 prospective studies, 13 retrospective studies, and 2 cross-sectional studies. Seven studies described data from the Hunter Outcome Survey (HOS) registry [26,30,34,35,36,41,47]. Twelve studies included individuals younger than 18 months (with inclusion of patients from this age group unclear for one additional study); twenty-five studies included individuals aged from 18 months to 4 years; thirty-two studies included individuals aged from 5 years to 17 years; and twenty studies included individuals aged at least 18 years (with inclusion of patients from this age group unclear for two additional studies). 

### 3.2. How Has IV Idursulfase Led to Improved Clinical or Patient-Centered Outcomes in Children and Adults with Diagnosed MPS II?

Overall, 20 studies assessed the impact of IV idursulfase on uGAG levels, 10 on cardiac outcomes, 10 on lung function outcomes, 15 on hepatosplenomegaly, 13 on 6MWT outcomes, 10 on joint function outcomes, 12 on growth outcomes, 2 on patient-reported outcomes, and 21 on safety and immunogenicity outcomes. 

An overview of the impact of IV idursulfase on key outcomes by treatment duration is shown in Table 3.

#### 3.2.1. uGAG Levels

Levels of uGAGs have been shown to decrease after ERT in patients with MPS II in conjunction with improvements in clinical outcomes; therefore, it has been suggested that uGAG levels could serve as a partially predictive biomarker to assess treatment efficacy [13,49]. Evidence from RCTs showed that treatment with IV idursulfase significantly reduced uGAG levels after 12 months of treatment in children and adults with MPS II (range of the change from baseline: −43% to −52.5%, *p* < 0.0001) [13,19]. An open-label extension showed a 77.4% reduction in uGAG level from baseline following 36 months of treatment (91/94 patients had uGAG levels above the upper limit of normal [ULN; 127 μg/mg creatinine] at baseline and 31/94 patients had uGAG levels above the ULN at 36 months) [11]. Similarly, a pediatric, phase 4, clinical study reported a 54.4% reduction in uGAG levels after 12 months of treatment [12]. In a clinical trial in adults only (mean [range] age: 30.1 [21.1–53.9] years), a 79.9% reduction in uGAG levels was reported after 12 months of IV idursulfase treatment, with all patients reporting normal values by the end of the study at 12 months [22]. 

Prospective observational studies in children and adults support the findings from the clinical trials but reported relatively higher reductions in uGAG levels after 3–5 years of treatment (range: 70.5–74.6%) [29,30,33]. Retrospective pediatric studies with follow-up periods ranging from 12 months to 10 years also demonstrated significant [36,38,43] or numerical [44,45] reductions in uGAG levels with IV idursulfase (Table 3). In studies with a duration over 12 months, increases in uGAG levels were described in two retrospective studies, with a total of seven and two patients, respectively [40,45]. The increases were attributed to high anti-drug antibody titers [40,45], although this was not consistently reported. Another study showed that reduction in uGAG level was significantly affected by neutralizing antibody status [21]. Short-term studies (4–12 months) reported decreases in uGAG levels over time in response to IV idursulfase [11,12,13,19,22,33,38].

#### 3.2.2. Cardiac Outcomes

Short-term clinical and observational studies showed statistically non-significant improvements in left ventricular mass index (LVMI) after 12 months of treatment with IV idursulfase in both children and adults with MPS II [13,22]. However, statistically significant improvements in LVMI were reported in children after 2–5 years of treatment, with numerical reductions reported in other cardiac parameters including aortic morphology and mitral and tricuspid valve function [28,42] (Table 3). Furthermore, LVMI was improved or sustained in patients who had cardiac hypertrophy at baseline [13,22,30,33,45]. Despite improvements in LVMI, progression of valve disease occurred in studies with observation periods ranging from 5 years to 14 years [33,45]. One study, however, showed an improvement or stabilization of valve regurgitation (particularly mitral and tricuspid valves) after 3.5 years of treatment [29].

#### 3.2.3. Respiratory Outcomes

Evidence from RCTs showed that treatment with IV idursulfase over 12 months resulted in small, statistically non-significant improvements in percentage predicted forced vital capacity (FVC) in children and adults with MPS II [13,19,22]. Minimal changes in absolute FVC and percentage predicted FVC were sustained over 3- and 8-year observation periods [11,43], with a decrease reported over a 10-year period [45]. Across studies, larger improvements were more likely to be reported with absolute FVC than with percentage predicted FVC, which may reflect the potential impact of growth in young patients during treatment with idursulfase [11,45]. 

#### 3.2.4. Hepatosplenomegaly

Prospective clinical studies demonstrated a statistically significant [13,19,22] or numerical [12] reduction in both liver size and spleen size with 12 months of IV idursulfase treatment (Table 3). In pediatric populations, reductions in liver volume and spleen volume of 17.4% and 20.6%, respectively, were reported after 12 months of treatment [12], whereas reductions of 33% and 31%, respectively, were reported in adults [22]. Significant [11,33] or numerical [30,40] improvements were maintained over 3–8-year observation periods in children and adults (Table 3).

#### 3.2.5. 6MWT Outcomes

In clinical trials, increased distances walked in the 6MWT were observed after 12 months of treatment [13,19,22], with the largest change observed in adults (Table 3) [22]. Similarly, over 3 years of treatment in an open-label clinical trial extension, the largest increase in 6MWT results was observed in adults (48.0 m), whereas children younger than 12 years and those aged between 12 years and 18 years reported smaller increases of 8.0 m and 0.7 m, respectively [11]. In longer-term observational studies ranging from 3 years to 10 years, changes in 6MWT results ranged from 41.0 m to 89.0 m [30,33,43,45], with the changes varying depending on age and disease severity, as discussed in the following section.

#### 3.2.6. Joint Range of Motion Outcomes

Short-term (<12 months) and long-term (range 4–8 years) ERT demonstrated limited efficacy on joint range of motion (JROM) outcomes regardless of disease severity and age [22,25,33,40], potentially reflecting limited delivery of ERT to the joints.

#### 3.2.7. Growth Outcomes

Benefits on growth outcomes following treatment with IV idursulfase in children with MPS II has not been consistently reported. Clinical studies in pediatric populations showed that patients who were prepubertal or younger than 12 years of age had a similar growth velocity (4.3–8.1 cm/year) to that of age-matched general populations after 1–3 years of IV idursulfase treatment [12,36]. Retrospective and cross-sectional observational studies [39,46,47] demonstrated height improvement with up to 3 years of IV idursulfase treatment across different pediatric age groups (duration of ERT treatment was not stated in Patel et al. [46]). However, a prospective observational study by Żuber et al. did not see any significant difference in the course of average growth in 13 ERT-treated children over 1–5.5 years of treatment compared with that in a retrospective ERT-naive MPS II cohort [31]. Nevertheless, it should be noted that a neuronopathic phenotype was present in all but one patient in this study, whereas across the aforementioned studies it was present in 0–80% of patients (where reported) [12,36,39,46,47].

#### 3.2.8. Patient-Reported Outcomes

Only two of the identified studies assessed patient-reported outcomes, highlighting the need for additional data of this nature [11,33]. In a phase 2/3, long-term, open-label extension study, functional status on ability to perform daily physical activities was assessed using the self-reported Child Health Assessment Questionnaire Disability Index (CHAQ DIS) in children older than 12 years, with scores ranging from 0 (no problem) to 3 (unable to perform). Statistically significant improvements from baseline in overall disability score were observed at several time points up to 3 years; after 24 months, CHAQ DIS score (mean ± standard error) had improved, with a change from baseline of −0.15 ± 0.65 (*n* = 44, *p* = 0.031) [11]. Statistically significant improvements were also observed in the hygiene (−0.3 ± 0.12) and reach (–0.4 ± 0.13) domains after 24 months [11]. In an observational study, the change in Mucopolysaccharidosis Health Assessment Questionnaire scores was assessed after 5–9 years of IV idursulfase in 15 patients with MPS II; improvements were reported in 23 of 52 questionnaire items in patients with non-neuronopathic MPS II (worsening in 3 of the 52 items), whereas improvements in only 9 of the 52 items were shown in patients with neuronopathic MPS II (worsening in 32 of the 52 items) [33].

#### 3.2.9. Adverse Events

After 12 months of IV idursulfase treatment in controlled trial settings, the majority of patients had at least one adverse event (AE); most AEs were mild or moderate in severity [12,19,22]. Serious AEs were reported in 20.0–46.4% of patients, and the majority of these were considered unrelated to treatment [12,19,22]. The most commonly reported AEs over 1 year of treatment included pyrexia, headache, pharyngitis, upper respiratory tract infections, rhinitis, abdominal pain, urticaria, and vomiting [12,19,22]. AEs possibly related to treatment were commonly associated with the infusion of the medication [12,19,22]. In clinical and observational studies, after 12 months of IV idursulfase treatment, 31.7–68.8% of patients reported infusion-related reactions (IRRs) [12,19,34], with fewer IRRs reported in real-world settings than in clinical trial settings. Of note, the incidence of IRRs was highest during the first 3 months after treatment administration and decreased thereafter [19,22,34], and IRRs were similar in nature and severity to those reported with placebo [19]. Broadly, long-term evaluations of IV idursulfase (2–8 years of follow-up) revealed no additional safety signals (Table 3) [11,30,33,37,40,43]. After 2–3 years of treatment in an open-label, phase 2/3 extension study, the percentage of patients who experienced an IRR was less than that reported at the 3-month peak time point for IRRs during treatment (<5% vs. ~40%) [11]. In addition, in two long-term observational studies (5–7 years of treatment), only 4 of 22 patients in one study [40] and 4 of 17 patients in the other [33] reported IRRs, suggesting an overall decreasing trend in IRR occurrence over time.

#### 3.2.10. Immunogenicity

Immunogenicity in response to IV idursulfase was assessed in 15 studies [11,12,13,19,20,21,22,26,27,34,36,38,40,43,45] (Table 3). Across these studies, 12.1–69.5% of patients tested positive for immunoglobulin G anti-drug antibodies (ADAs) after treatment, and no clear differences based on treatment duration or age were found [27,43]. The development of neutralizing ADAs was less common (range: 15.9–53.6%) [11,12,20,34]. Although the presence of ADAs may be associated with a reduced efficacy of IV idursulfase at lowering uGAG levels [21,40,45], clinical efficacy and safety outcomes were rarely affected [20,21,40,43]. Studies indicated that treatment response may show higher correlations with neutralizing ADA titers and genotype than with uGAG response [21], but this warrants further evaluation.

### 3.3. What Are the Limitations of Using the 6MWT as an Isolated Clinical Indicator?

Completion rates of the 6MWT in two 2-year observational studies demonstrated that conducting the test in patients with MPS II may be challenging in clinical practice, with only 2 of 27 patients completing the test in one study [37] and 4 of 11 patients in another [38]. Although improvements were observed in some of these patients, the low numbers prevent any firm conclusions from being drawn. Importantly, several long-term studies highlighted the difficulty of conducting 6MWT assessments, which may cast doubt over the robustness of using the 6MWT as an isolated treatment indicator for patients with MPS II. The 6MWT was rarely used in the study of Tomanin and colleagues (the test was performed in only 6 of 27 participants), because patients were not compliant or were not able to perform the test, particularly in the youngest age group (≤5 years of age) [29]. The 6MWT could not be properly conducted in 20 of 45 patients with MPS II from Poland (5–20 years of age) because they were not able to walk independently or were unable to cooperate owing to intellectual disability [32]. In a study by Parini et al., most of the patients had neuronopathic MPS II (11/17 patients, 2.3–25.5 years of age) and could not complete the test owing to hyperactive behavior or because they used a wheelchair for mobility [33]. Correlational analyses between 6MWT and other clinical outcomes were not conducted in the identified studies. The studies suggested, however, that the large variance observed in 6MWT results, particularly across age groups, may be attributable to changes in growth [38]. Therefore, using 6MWT results as an isolated outcome without context from other clinical parameters may not be appropriate.

### 3.4. What Are the Short- and Long-Term Benefits of the Use of IV Idursulfase?

Across the identified studies, the earliest benefits consistently reported with IV idursulfase in patients with MPS II were improvements in uGAG levels and hepatosplenomegaly. An overview of the short- and long-term benefits of IV idursulfase on key outcomes by treatment duration is shown in Table 3. Clinically relevant improvements were demonstrated within the first 3–4 months of treatment for both adults and children [11,12,19,22]. Glamuzina and colleagues also described a trend toward a rapid reduction in uGAG levels over the first 8 weeks of treatment. After this early reduction, improvements were sustained for both uGAG and hepatosplenomegaly outcomes over evaluation periods ranging from 1 year to 8 years [38].

In children and adults, improvements in cardiac and lung outcomes were reported as early as 12 months after treatment with IV idursulfase; however, these changes were not statistically significant [13,19,22,42]. Both outcomes required longer evaluation periods to demonstrate benefits of IV idursulfase, as confirmed by statistically significant and clinically relevant reductions in LVMI, which were reported at 2 years after starting treatment and were maintained for up to 8 years during follow-up [28,30,33,37,42]. For lung outcomes, although trends for improvement were reported after 12 months of treatment, statistically significant changes were not observed [11,30,38,43,45]. Some studies reported minimal changes in FVC (percentage predicted) after 2–8 years of treatment (Table 3), suggesting stable respiratory function with long-term treatment [11,38,43]. 

The early effect of IV idursulfase on 6MWT results was shown in the phase 2/3 pivotal RCT, which reported a significant improvement after only 4 months of treatment [11]. A statistically significant improvement in 6MWT results (an increase of between 44.3 m and 47.0 m in distance walked) was consistently reported across RCTs as early as 12 months after treatment in children and adults [13,19]. A similar level of improvement was maintained after 3 years of treatment, with a peak after 20 months (+42 m), although this effect was not equally distributed across age groups. Patients older than 18 years showed the largest improvement (+48 m), which potentially drove the observed overall effect (see Section 3.5) [11]. Studies with 7–10 years of follow-up reported a continued improvement in 6MWT results in non-neuronopathic patients (from +67.0 m to +89.0 m), although the range of effect was wide (from −129.0 m to +292.0 m) [33,43,45]. There were limited long-term data in neuronopathic patients, most likely due to the challenge of conducting the 6MWT in this population, as discussed in Section 3.3.

IRRs are a concern for patients starting IV idursulfase treatment; however, studies appeared to show a declining trend in the incidence of IRRs over time [33,34,36,40]. Based on HOS data, most IRRs were reported in the first 3 months (26 of 33 patients with IRR events), with only 5 patients reporting IRRs after 3 months, 2 of whom reported IRRs after 6 months [34]. Similarly, in the phase 2/3, open-label extension trial, over 3 years of treatment IRR incidence peaked at 3 months and declined to less than 15% between 6 months and 18 months, and less than 10% between 2 years and 3 years [11].

Long-term IV idursulfase was shown to reduce the risk of death in patients with MPS II, although this was more pronounced in patients with non-neuronopathic MPS II [41]. Long-term ERT treatment (~4.1 years) was associated with a 54% lower risk of death than no treatment; however, the risk of death in patients with MPS II was fivefold higher in those with cognitive disability than in those without [41]. Increased survival with IV idursulfase treatment was demonstrated in a large population of patients with MPS II: median survival based on Kaplan–Meier estimates (95% confidence interval) was 33.0 (30.4, 38.4) years in treated patients and 21.2 (16.1–31.5) years in untreated patients, confirming the long-term benefits of ERT in patients with MPS II [41]. A post-marketing study in Japan showed a relatively high survival rate at 7 years in patients with neuronopathic MPS II, although the survival rate was lower than that in non-neuronopathic patients (76.7% vs. 91.2%) [43]. 

### 3.5. Are the Benefits of Treatment with IV Idursulfase Based on Presymptomatic Diagnosis, Phenotype, or Age?

#### 3.5.1. Presymptomatic Diagnosis

A limited number of the identified studies evaluated patients with presymptomatic diagnosis [28,32]. Two patients were identified whose disease was diagnosed prenatally and for whom outcomes after IV idursulfase treatment were reported [28,32]. In these patients, over 3 years of treatment, cardiac outcomes improved, whereas liver and 6MWT outcomes were sustained. One patient who was 5 years of age at baseline assessment had non-neuronopathic MPS II diagnosed early owing to a family history of MPS II, and therefore initiated IV idursulfase between birth and 3 months of age. This patient had no abnormal cardiac signs at baseline assessment (at 5 years of age) or after 3 years of follow-up; furthermore, from baseline to 3 years of follow-up, liver size remained in the normal range and the distance covered in the 6MWT improved from 400.0 m to 440.0 m. Cognitive ability was considered ‘normal’ at baseline assessment and at the 3-year follow-up visit [32]. Cardiac outcomes for another patient whose disease was diagnosed prenatally were reported by Brands and colleagues. After 3 years of follow-up (ERT was started at 1 year of age), improvements in interventricular septum thickness in diastole (IVSd) and LVMI Z-scores were reported (IVSd: 2.88 at baseline, −0.56 at 3 years; LVMI: 0.10 at baseline, −0.03 at 3 years) [28].

#### 3.5.2. Phenotype

Patients with neuronopathic MPS II were excluded from the pivotal RCTs evaluating the efficacy and safety of IV idursulfase; therefore, differences in treatment benefits between patients with neuronopathic and non-neuronopathic MPS II are based solely on observational findings (Table 2). Two studies evaluated outcomes with ERT exclusively in patients with neuronopathic MPS II [40,42] and reported improved somatic symptoms with long-term treatment with IV idursulfase over 5 years [40,42]. In the first study, hepatosplenomegaly and frequency of respiratory infections were reduced in all 22 patients, and JROM improved in 7 of 21 patients; joint disease and cardiac disease were stabilized in 13 of 21 patients and 19 of 22 patients, respectively. Reductions in uGAG levels ranging from 22% to 97% were reported in 20 of 22 patients [40]. In the second study, a statistically significant reduction in LVMI, from 70.9 g/m^2^ to 26.8 g/m^2^ (*p* = 0.003), was observed after 5 years of treatment with IV idursulfase [42].

Data on mixed populations comprising patients with neuronopathic MPS II and patients with non-neuronopathic MPS II were reported in 17 studies. Across these studies, 44–92% of patients had neuronopathic MPS II [25,26,27,29,30,31,33,34,35,36,37,38,41,43,44,46,47]. Results across these populations were often pooled, and as a result not all studies presented results based on phenotype. Therefore, caution is needed when interpreting the results of these studies because they reflect phenotypically heterogeneous populations. After 2–8 years of treatment with IV idursulfase in studies in patients with neuronopathic MPS II, improvements were commonly observed in uGAG levels and hepatomegaly [29,33,36,38,40]; other outcome improvements (e.g., for growth, splenomegaly, or cardiac disease) were reported but require further evaluation [29,33,37,42,47]. In a 24-month HOS analysis, a significant improvement in growth was observed in children aged 8–15 years, which was significantly less pronounced in patients with genotypic variants associated with neuronopathic MPS II (deletions, large rearrangements, and nonsense variants) than in patients with other variants. However, cognitive involvement was not found to be related to the growth deficit or to the response to treatment [47]. An analysis in children aged 3–6 years with neuronopathic MPS II showed no effect on growth up to 5.5 years after starting treatment [31]. 

Another study described significant reductions in uGAG levels after 1 year and 3 years of treatment in patients with neuronopathic MPS II, whereas a significant reduction was only observed after 3 years of treatment in patients with non-neuronopathic MPS II [29]. In addition, after 3 years of treatment, a greater proportion of patients with neuronopathic MPS II than of those with non-neuronopathic MPS II reported positive outcomes (disease improvement or stabilization) in hepatomegaly (60% vs. 50%), splenomegaly (76.9% vs. 55.6%), otological disorders (58.3% vs. 33.3%), and adenotonsillar hypertrophy (75% vs. 20%) [29]. The authors noted that this could potentially be explained by the presence of more advanced clinical signs in neuronopathic patients at the start of treatment, for whom an improvement due to ERT could be more evident than in those with non-neuronopathic MPS II [29].

Poor efficacy in JROM outcomes was observed over 1–4 years of treatment with IV idursulfase regardless of disease phenotype [25,29]. As mentioned previously, studies evaluating 6MWT in patients with neuronopathic MPS II highlighted the difficulty of examining this population, because the patients were found to have limited ability to perform the test [29,32,33,43]. Accordingly, the benefit of IV idursulfase on 6MWT results was mostly restricted to patients with non-neuronopathic MPS II. 

A genotype analysis of patients aged 5 years or older with non-neuronopathic MPS II who received IV idursulfase for approximately 2 years found that patients with nonsense or frameshift variants may be more likely than those with missense variants to develop antibodies, to experience IRRs, and to have a reduced uGAG response [20]. This study, however, did not include patients with neuronopathic MPS II or patients younger than 5 years.

#### 3.5.3. Age

All identified studies showed a consistent reduction in uGAG levels in response to IV idursulfase regardless of age group. Complete normalization of uGAG levels was observed only in a single study conducted solely in adults [22], whereas across other age groups, uGAG normalization was not universally achieved or reported. In contrast with the findings from the study in adults, Ueda et al. reported less of a reduction in uGAG levels in patients aged 15 years or older than in those younger than 15 years [43]. Furthermore, they concluded that the degree of clinical improvement after 8 years of IV idursulfase treatment, with clinical improvement defined as improvements in skin condition, joint mobility, and respiratory symptoms including obstruction, apnea, recurrence of airway infections, and worsening of respiratory failure, was similar between age-stratified subgroups (<15 years vs. ≥15 years) [43]. The authors noted, however, that in patients with non-neuronopathic MPS II, children had better results than adults in the 6MWT and for forced expiratory volume in the first second (FEV_1_), but that variability in other background characteristics may have confounded this observation [43]. Tomanin and colleagues evaluated cardiac valve disease after 3.5 years of treatment in three age groups: 5 years or younger, older than 5 years and 12 years or younger, and older than 12 years. Most patients across these age groups had improved or stabilized mitral valve regurgitation. Patients who were 5 years of age or younger experienced a worsening in aortic valve regurgitation but stabilization in tricuspid regurgitation, whereas patients older than 5 years had stable and worsened aortic and tricuspid valve regurgitation, respectively [29]. These benefits, however, are at odds with other studies, which showed a general progression in valvopathies despite IV idursulfase treatment [33,45]. 

Respiratory improvements appeared to be more pronounced the earlier treatment was started [36,45]. After 3 years of treatment, patients younger than 12 years and patients who were 12–18 years of age demonstrated sustained increases in absolute FVC (0.39 L and 0.45 L, respectively), whereas patients older than 18 years showed a decrease of 0.04 L [11]. Statistically significantly better lung function was reported after about 10 years of treatment in patients who started IV idursulfase early (before 8 years of age; *n* = 11; percentage predicted FVC of 69% [range: 34–86%]) than in those who started later (after 8 years of age; *n* = 13; percentage predicted FVC of 48% [range: 25–108%]) (*p* = 0.045) [45]. 

Hepatosplenomegaly was generally stable or improved with IV idursulfase over time regardless of age group [12,19,22,30,33,40,43]. However, Tomanin and colleagues only reported a significant improvement in hepatomegaly after 3.5 years of treatment in patients who were older than 5 years and 12 years or younger; similar improvements were not achieved in those aged 18 months or older and 5 years or younger, or in those older than 12 years [29]. In addition, no significant improvements in splenomegaly were reported in any age group in this study. The authors explained that these findings may have been related to the variable distribution of organomegaly at baseline. Across age groups, hepatomegaly was present in 58–100% of patients, whereas splenomegaly was present in 9–71% of patients; consequently, the relatively small number of patients with organ enlargement in some groups limited the sample size for inclusion in the analysis [29].

The improvement in height following 3 years of IV idursulfase treatment reported in a retrospective analysis was more pronounced in patients who started treatment when younger than 10 years (population age range: 6–19 years) than in those who started treatment at an older age [39]. In addition, the height benefit reported in the 24-month HOS analysis was greater in patients who started treatment at 8–11 years of age than in those who started treatment at 12–15 years of age [47].

Ueda and colleagues reported a greater improvement in 6MWT after about 7 years of treatment in patients aged 15 years or older than in those younger than 15 years (+55.0 m vs. −22.3 m in patients with neuronopathic MPS II), but this trend only held true for patients with neuronopathic MPS II (*n* = 11), among whom only one individual aged 15 years or older was included in the analysis. Conversely, in patients with non-neuronopathic MPS II (*n* = 15), the improvement in the younger patient group (<15 years, *n* = 7) was greater than that in the group aged 15 years or older (*n* = 8; +89.0 m vs. +27.9 m) [43]. In this study, 7 of the 138 patients in the safety analysis set underwent hematopoietic stem cell transplantation (HSCT) before ERT; no individual analysis was performed to evaluate differences in outcomes for this subgroup, so future research may be needed to evaluate effectiveness of ERT after HSCT.

Safety findings with IV idursulfase were generally consistent across age groups, with IRRs being commonly reported. Only three studies provided a breakdown of safety data by age, with a general trend toward more events in younger age groups than in older age groups. A 12-month analysis of HOS patients by Burton et al. showed that the incidence and time to first IRR were similar across different age groups (*p* = 0.427) [34]. Muenzer and colleagues reported that after 2 years of follow-up in HOS, the incidence of IRRs reported was higher in patients younger than 6 years than in patients aged 6 years or older (26.6% vs. 18.8% of patients [36]); details on serious IRR events were not reported. Serious AEs were reported in 12.9% and 20.2% of patients younger than 6 years and those aged 6 years or older, respectively [36]. According to Ueda and colleagues, after up to 8 years of treatment, 53.9% of patients younger than 15 years experienced at least one adverse drug reaction compared with 38.5% in patients aged 15 years or older; general disorders and administration-site conditions were reported in 23.6% and 13.5% of patients, respectively [43].

### 3.6. What Are the Benefits, If Any, of IV Idursulfase Treatment for Patients with Neuronopathic MPS II?

Of the seven identified studies reporting cognitive outcomes in patients with neuronopathic MPS II, all had a treatment duration of more than 2 years, highlighting the difficulty, particularly in the short term, in assessing cognitive outcomes in patients with MPS II [11,29,32,33,40,44,45]. After about 5–6 years of treatment, cognitive ability was mostly worsened or maintained, regardless of cognitive level or of age when starting treatment [29,32,40,44]. In a case series study, 2 (9%) of 22 patients with neuronopathic MPS II experienced a slight improvement in cognitive ability [40]. However, both patients were evaluated by investigator impression, which the authors noted may have been a reflection of improvements in other domains (e.g., sleep, breathing) rather than drug effects on cognitive ability per se [40]. Furthermore, in another study in patients with neuronopathic MPS II (2–16 years of age), patients were not testable due to a fast decline in cognitive ability despite 3–8 years of ERT [33]. A negligible effect on cognitive outcomes with IV idursulfase is expected, given that it is unable to cross the BBB [6]. Nevertheless, patients with neuronopathic MPS II may still benefit from the positive effect of IV idursulfase on somatic outcomes.

### 3.7. Are There Any Benefits or Disadvantages Associated with the Use of IV Idursulfase in Patients Aged Younger than 18 Months or Younger than 5 Years?

A summary of outcomes with IV idursulfase in patients younger than 18 months is shown in Table 4. Five studies showed a reduction in uGAG levels with IV idursulfase in pediatric populations that included children younger than 18 months [12,36,38,43,45], with statistical significance reported in three of them (Table 4). However, the results were not specific to this age group, and the pediatric populations represented a small proportion of the overall study populations. In the study populations, in the three studies showing significance, 4 of the 28 patients were younger than 2 years, 3 of the 11 patients were younger than 18 months, and 11 of the 124 patients were younger than 12 months of age [12,36,38]. One other study may have included patients younger than 18 months of age, but the article did not provide enough clarity regarding age distribution to draw any conclusions [30].

Cardiac outcomes were not reported in patients younger than 18 months across studies with a treatment duration of 6–12 months; however, one study included a single patient whose age was in this range; MPS II was diagnosed prenatally for this patient, who started IV idursulfase treatment at between 3 months and 12 months of age. Improvements in cardiac outcomes were observed after 3 years of treatment in this study [28]. Similarly, lung function was not specifically reported in patients younger than 18 months. Moreover, one article remarked that the single patient younger than 18 months included in the study population was too young (14 months) to perform lung function tests [38]. 

Two of the five pediatric studies that included patients younger than 18 months showed improvements in hepatosplenomegaly after 3–6.5 years of treatment; however, the exact number of patients in this age group was not reported (Table 4) [12,43]. Overall, five of 21 safety assessments included patients younger than 18 months [12,21,30,38,43]. However, these did not provide safety results specific to this age group (Table 4). 

Two studies were identified that may provide insights into the effect of IV idursulfase in patients who started treatment when aged younger than 5 years. In a 12-month, open-label, prospective study by Giugliani et al., 20 (71%) of the 28 patients were younger than 5 years of age [12]. In the 3-year, HOS analysis by Muenzer et al., 310 (49%) of the 639 patients were aged 5 years or younger [30]. These two studies did not stratify their results by age, but we may be able to infer that the benefits reported by these studies, such as decreases in GAG levels, reductions in liver and spleen volumes, and improvements in FVC and LMVI (Table 3) also apply to children younger than 5 years old, given that they represented a substantial proportion of the study population. A genotype analysis conducted by Pano and colleagues that enrolled 28 patients who were 5 years of age or younger at screening showed that the incidence of IRRs was higher in patients with complete deletion/large rearrangements (87.5%) than in those with frameshift/splice site variants (50%) or missense variants (46.2%). Reduced responses in liver volume and uGAG levels were also reported in patients with complete deletion/large rearrangements compared with those with missense variants [21]. Although seroconversion was significantly associated with complete deletion/large rearrangements and frameshift/splice site variants compared with missense variants (100% vs. 31%), the authors showed that following adjustment for genotype, the risk for IRRs was driven by genotype, not antibody status, whereas efficacy outcomes were driven by both genotype and the presence of neutralizing antibodies [21]. In the 3.5-year, observational, prospective study by Tomanin et al., 13 (48%) of the 27 patients were 5 years of age or younger [29]. In this age group, uGAG levels were significantly reduced after 1, 2, and 3 years of IV idursulfase, and mitral valve regurgitation was improved or stabilized after 3 years of treatment; however, no significant improvements were reported for hepatosplenomegaly or JROM. 6MWT results were improved by 20% in 3 of 13 patients; not all patients were tested owing to challenges in conducting these tests in very young patients [29]. 

### 3.8. What Are the Outcomes with Different Doses of IV Idursulfase?

The majority of identified studies evaluated the effect of IV idursulfase administered every week at the recommended clinical dose of 0.5 mg/kg; therefore, there is limited evidence regarding the use of IV idursulfase at other doses. In the clinical trial publication authored by Muenzer et al., different dosages of IV idursulfase were explored, including 0.5 mg/kg administered every other week (EOW) [19]. In the first phase 1/2 trial to evaluate IV idursulfase, Muenzer et al. administered dosages of 0.15, 0.5, or 1.5 mg/kg EOW. Results were pooled and showed an improvement in somatic outcomes with no clinically relevant differences across the three dosages [13]. Overall, these RCTs demonstrated that weekly dosing was more effective than the EOW regimen [13]. In one study, all patients received ERT at 1 mg/kg weekly, although the reason for this relatively higher dosage regimen was not explained [46]. This study evaluated the impact of IV idursulfase on growth in 26 patients who started treatment at a mean age of 4.5 years. Compared with untreated patients, IV idursulfase was shown to improve height and weight but only in children older than 8 years, suggesting that a long-term evaluation period (treatment duration was not reported) is required to assess growth in patients with MPS II [46]. 

## 4. Discussion 

Overall, this SLR identified 33 articles describing analyses regarding ERT with IV idursulfase for the treatment of MPS II. Among these, 26 articles described observational studies, with the remainder reporting RCTs and/or open-label clinical trials. The findings of this SLR help to address several questions on the efficacy, effectiveness, and safety of IV idursulfase, particularly regarding the short- and long-term benefits of treatment and the effect of treatment in patient subgroups. 

Overall, IV idursulfase consistently demonstrated improvements in somatic outcomes in both children and adults, with benefits seen as early as 4 months after treatment and maintained over long follow-up periods of up to 10 years. Self-reported patient outcomes, however, were not widely evaluated. Only two studies reported this type of outcome [11,33]. It remains important to obtain the perspective of the patient (or caregiver as proxy) when evaluating the effectiveness of a treatment, although self-evaluation may not be possible in patients with neuronopathic disease. Clear early benefits of IV idursulfase were noted in relation to uGAG levels and hepatosplenomegaly, with reductions observed in the first 2–4 months of treatment and sustained over long-term observational periods of up to 8 years [11,12,13,19,22,24,29,30,32,33,38,40,43]. A few studies showed an increase in uGAG levels in some patients receiving long-term ERT treatment [40,45], which may be attributed to ADAs, although this was inconsistently reported. One study, however, showed no improvement in hepatosplenomegaly in children who were 5 years of age or younger after 3.5 years of treatment regardless of age at initiation of ERT [29]. The authors suggested that this may be partially explained by the relatively small number of patients meeting the analysis criteria in this study. Pano and colleagues showed in children that variable treatment responses in organ volume and uGAG levels may be underpinned by genotype, because a reduced response was shown to correlate with substantial genomic alterations (e.g., complete deletions) [21]. 

Studies consistently showed a benefit of IV idursulfase on LVMI, with a trend toward improvement in the 12 months after initiation, and this result was supported by statistically significant improvements found after up to 5 years of treatment [28,33,42]. Nevertheless, this was not the case for other cardiac parameters (e.g., valve disease), in which the treatment effect remains unclear. Changes in respiratory outcomes were minimal in most short- and long-term studies, suggesting IV idursulfase helps to sustain baseline lung function without any major improvements. A 10-year study, however, showed that earlier initiation of ERT (before vs. after 8 years of age) may significantly improve respiratory outcomes compared with starting treatment later during the disease course [45]. Care should be taken when interpreting these respiratory outcomes, because although significant changes in absolute FVC were reported, changes in percentage predicted FVC were minimal; the former outcome may not be as informative as the latter, because it does not account for differences in, and the influence of, patient growth on lung function. 

Improvements in 6MWT results were reported after 12–36 months of treatment, with greater benefit observed in adults than in children. Improvements were also observed after 3–10-year follow-up periods. However, several real-world studies highlighted the difficulty in conducting this evaluation, particularly for the youngest patients and those with intellectual and physical disabilities. Accordingly, few patients across studies were able to undertake the 6MWT, indicating that 6MWT results in isolation may not be reliable efficacy metrics in the youngest or most severely affected patients with MPS II. Moreover, studies suggest that improvement in 6MWT results may be driven by changes in growth parameters, thus confounding the interpretation of 6MWT data. 

Safety findings were consistent with what would be expected for populations with MPS II. Notably, studies showed a declining trend in the incidence of IRRs over time with a peak after 3 months of treatment and a decline thereafter [11]. After 5–7 years of treatment, a low incidence of IRRs was maintained regardless of disease phenotype. Most IRRs were hypersensitivity reactions that were either allergic or nonallergic reactions and were generally resolved by interrupting or reducing the infusion rate and/or by the administration of antihistamines, antipyretics, and/or corticosteroids [36]. When premedication or infusion-rate reductions were ineffective for preventing IRRs, desensitization was shown to be successful [50]. Although marginal, the incidence of IRRs appeared to be higher in patients younger than 6 years than in those aged 6 years or older [36]. A similar trend was observed when safety was analyzed using a younger than 15 years or 15 years or older cut-off [43]. Differences in IRRs may also be driven by genotype: Pano and colleagues showed that IRR incidence was highest in patients with complete deletions/large rearrangements compared with those with other variants [21]. Although the development of ADAs was observed in 28.6–60.0% of patients across studies, it did not appear to correlate with a reduction in efficacy or a change in the safety profile (after adjustment for genotype). Consistent with the treatment benefits reported, a long-term survival study showed that treatment with IV idursulfase was associated with a 54% reduction in risk of death compared with no treatment [41]. A relatively high survival rate was also reported in patients with neuronopathic MPS II, thus supporting the long-term use of IV idursulfase regardless of disease phenotype [43]. 

Overall, age subgroups (infants, preschool children, school children, and adults) were represented to a similar degree across studies, except for infants younger than 18 months. Of the 33 identified studies, 12 included patients younger than 18 months. These studies showed the potential benefits of IV idursulfase, although the low patient numbers preclude drawing firm conclusions. ERT treatment in infants with MPS II has also been described in case studies, which were not captured by our search. One such case study describes treatment with IV idursulfase that was started at approximately 6 months of age in a boy presenting with postnatal respiratory distress. After 6 weeks of treatment, improvements in respiratory outcomes (oxygen requirement, respiratory rate, upper airway obstruction symptoms, and snoring) were observed, supporting the use of IV idursulfase early in the disease course [51]. Lampe and colleagues described a case series of eight patients with MPS II for whom idursulfase treatment was initiated at under 1 year of age; all of the patients treated for more than 6 weeks showed improvements and/or stabilization of some somatic manifestations while on treatment, with no new safety concerns observed. Moreover, in some cases, caregivers reported that early-treated patients experienced a less severe clinical course compared with other affected family members [52]. Data were reported for two patients whose disease was diagnosed prenatally and for whom there were reported outcomes after IV idursulfase treatment in two articles identified in our search. Over 3 years of treatment, cardiac outcomes improved whereas liver and 6MWT outcomes were sustained [28,32]. Early disease detection and treatment prior to the onset of clinical symptoms are also discussed in a study comparing the disease course of two siblings who started receiving IV ERT within a month of diagnosis owing to clinical disease or before disease was evident, respectively. Long-term (8–8.7 years of ERT treatment) findings from this sibling pair showed improved somatic and neurocognitive outcomes in the brother receiving presymptomatic treatment, confirming the importance of early initiation of therapy [53].

To explore further the benefits of IV idursulfase in young children, we examined studies that included patients younger than 5 years. Across three pediatric studies [12,29,30], the proportion of patients younger than 5 years ranged from 48% to 71%, thus comprising most patients. Overall, these studies showed a consistent improvement in uGAG levels over 1–3 years of treatment but with conflicting findings regarding treatment effect on hepatosplenomegaly. A small case series in children younger than 5 years (*n* = 6), not included in this SLR, further supports the benefit of early IV idursulfase. Improvements in several outcomes were reported after 8 months of IV idursulfase treatment, although not in all patients: reduced uGAG levels were observed in five patients, reduced spleen size in two, and reduced liver size in one [54]. These limited findings support the early use of ERT, but further evidence in very young patients and in those with presymptomatic diagnosis is needed. The addition of MPS II to the US Recommended Uniform Screening Panel in 2022 will help to drive earlier detection and treatment initiation [16]. With MPS II newborn screening programs becoming more widely implemented across large populations in US states, a better understanding of the incidence of MPS II in the USA is expected. 

Only two studies exclusively evaluated treatment response in patients with neuronopathic MPS II [40,42]. Most other studies included a mixed population characterized by diverse degree of CNS involvement, thus warranting caution when drawing conclusions regarding treatment benefits based on disease phenotype. Overall, although somatic improvements were observed with IV idursulfase administration in patients with neuronopathic MPS II (mostly in terms of uGAG levels and liver volume), these were generally not as pronounced as those in patients with non-neuronopathic MPS II. As might be expected, cognitive outcomes were only assessed in long-term studies and findings were inconclusive, aligning with the limited efficacy of conventional ERT to address symptoms involving the CNS. A prospective observational study that enrolled boys with MPS II aged between 2 and 18 years who were receiving IV idursulfase showed that neurodevelopmental changes in individual patients with MPS II followed highly variable trajectories in the progression of disease, confirming findings from the studies identified in our search [55]. Reported improvement in cognitive outcomes may have resulted from improvements in other parameters (e.g., respiratory parameters), which may have exerted positive change in environmental interactions and behavior as opposed to a direct effect on the brain. Alternative approaches to manage neuronopathic MPS II are emerging, including the use of gene therapy and fusion proteins [3,14]. 

Marked phenotypic heterogeneity in patients with MPS II may confound the identification of correlations between treatment outcomes and phenotype. Genotype–phenotype analyses have been conducted in an attempt to explain the variation in disease phenotype and its influence on treatment outcomes [20,21,56]. Deletion, frameshift, and nonsense variants have been shown to be associated with neuronopathic MPS II and a reduced treatment response [20,21,56]. However, missense variants, which are the most identified *IDS* variants in MPS II, can be associated with both neuronopathic and non-neuronopathic phenotypes [56]. The complex relationship between genotype and phenotype probably underpins the variation reported in treatment outcomes across different study populations, and thus warrants further study.

### Strengths and Limitations

This was a comprehensive SLR capturing relevant literature on the clinical efficacy and effectiveness of IV idursulfase in patients of all ages with MPS II, designed and conducted using robust methodology in accordance with the 2020 PRISMA guidelines. The time frame used for the systematic electronic searches of relevant publications (up to January 2023) may have excluded pertinent studies published after this date. As more data become available for novel MPS II therapies (e.g., gene therapy, fusions proteins), it will be important to conduct equally robust searches to help to inform the overall management of MPS II. A quality assessment of the literature was performed to account for risk of bias; however, differences in study designs and evaluation metrics preclude robust comparisons between studies. As is typical of real-world studies on rare diseases with no existing therapeutic alternative, there was a lack of comparators in most of the identified literature. Owing to the small number of people with MPS II, very young age groups were not represented equally across studies. Moreover, data on the effect of different dosages of IV idursulfase on treatment outcomes were insufficient to draw any meaningful conclusions.

## 5. Conclusions

This SLR discussed the evidence regarding the efficacy, effectiveness, and safety of ERT with IV idursulfase for MPS II in a broad spectrum of patients. The identified studies showed that, overall, IV idursulfase treatment results in short- and long-term improvements of several clinical and patient-centered outcomes. Safety findings were consistent with what would be expected for populations with MPS II. The benefits of IV idursulfase were dependent on phenotype, with improvements in clinical outcomes more pronounced in patients with non-neuronopathic MPS II than in those with the neuronopathic form. Nevertheless, it has been shown that IV idursulfase treatment resulted in some improvements in patients with neuronopathic MPS II because they still benefit from the positive treatment effect on somatic outcomes. Although participants in young age groups were limited in number and not represented equally across studies, it has been demonstrated that IV idursulfase treatment is associated with clinical outcome improvements, particularly when started early in life, and is accompanied by a favorable safety profile. The number of studies on the effect of different dosages of IV idursulfase on treatment outcomes is limited; therefore, no meaningful conclusions could be drawn. As more data become available on the natural history, progression, and potential novel therapies for MPS II, conducting robust SLRs will be important to help to inform the overall management of MPS II.

## Figures and Tables

**Table 1:** SLR eligibility criteria.

Category	Inclusion Criteria	Exclusion Criteria
Population	Patients with MPS II ○ Any age, phenotype, genotype, and stage of progression	Patients without MPS II Patients with another LSD
Intervention and comparator	IV idursulfase (Elaprase^®^)	IV idursulfase beta (Hunterase^®^) Intrathecal idursulfase
Outcome	Clinical efficacy, real-world effectiveness, and safety ○ Including but not limited to uGAG levels, liver volume, safety, 6MWT results, growth, lung function, cardiac function, cognitive outcomes, and long-term outcomes	HRQoL outcomes, economic burden, and other outcomes
Study design	Observational studies (including retrospective, cross-sectional, prospective, case-control, registry, cohort, and database studies) Clinical studies, any phase (RCTs and single-arm intervention studies) Meta-analyses	In vitro studies Animal studies Case studies
Publication type	Full articles and congress abstracts	Books, editorials, and letters
Language	Studies published in the English language	
Country	Not restricted by country or region	
Date	Not restricted by publication date	

6MWT—6-min walk test; HRQoL—health-related quality of life; IV—intravenous; LSD—lysosomal storage disease; MPS II—mucopolysaccharidosis type II; RCT—randomized controlled trial; uGAG—urinary glycosaminoglycan.

**Table 2:** Summary of included articles describing studies of IV idursulfase in patients with MPS II.

											Included Age Groups			
First Author andYear	Study Design	Country	Study Aims	Treatment Duration	Sample Size, *n* ^a^	Age at Diagnosis, Years	Age at Symptom Onset, Years	Age at Start of ERT, Years	Patients with Neuronopathic MPS II, Y/N (*n*/*N*)	Genotype Details (Y/NR)	<18 Months	18 Months to 4 Years	5 Years to 17 Years	≥18 Years
**Prospective studies**														
Bagewadi 2008 [23]	P, O, SC	UK	Safety of home ERT	15 (12–24) weeks ^b^	17	NR	NR	Range (4–15)	NR	NR		✓	✓	
Negretto 2014 [24]	P, O, SC	Brazil	uGAG and oxidative stress outcomes	6 months	12	NR	NR	Range (1.6–7.5)	NR	NR		✓	✓	
Muenzer 2007 [13]	P, RCT, SC	USA	Safety and efficacy	6–12 months	9 (Tx) 3 (Pbo)	NR	NR	14 (6–20) ^b^	N	NR			✓	✓
Giugliani 2014 [12]	P, OL, MC	Multiple countries	Safety, PK/PD, and efficacy	52 weeks	28	3.8 (0.2–6.5) ^e^	NR	4.0 (1.4–7.5) ^e^	NR	NR	✓	✓	✓	
Marucha 2022 [25]	P, O, SC	Poland	JROM	68–85 weeks (non-neuro) 230 (108–332) weeks (neuro)	16	3.3 (<1–8.5) ^b^	NR	8.7 (<1–28) ^b^	Y (12/16)	Y	✓	✓	✓	✓
Muenzer 2006 [19]	P, RCT, MC	Multiple countries	Safety and efficacy	53 weeks	32 (Tx) ^c^ 32 (Pbo)	5.2 (<1–20) ^b,d^	NR	15.1 (6.3–26.0) ^b,d^	NR	NR			✓	✓
Okuyama 2010 [22]	P, OL, MC	Japan	Safety and efficacy	12 months	10	7.9	NR	30.1 (21.1–53.9) ^b^	N	NR				✓
Giugliani 2017 [26]	P, O, MC, HOS	Multiple countries	Immuno-genicity	109 weeks	26	3.8 (0.1–20.0) ^e^	NR	NR	Y (13/24)	NR		✓	✓	✓
Kim 2013 [27]	P, O, SC	South Korea	Infusion-related allergic reactions	154 (13–180) weeks ^b^	34	NR	NR	12 (3–38) ^e^	Y (15/34)	NR		✓	✓	✓
Brands 2013 [28]	P, O, SC	The Netherlands	Cardiac outcomes	0–159 weeks	6	Prenatal to 6	NR	Range (1.0–10.8)	NR	NR	✓	✓	✓	
Muenzer 2011 [11]	P, OL, MC	Multiple countries	Long-term outcomes	2–3 years	94	NR	NR	14.5 (5.4–30.9) ^e^	NR	NR			✓	✓
Tomanin 2014 [29]	P, O, MC	Italy	Effectiveness	~3.3 years	27	3.5 (0.9, 15.5) ^e^	NR	5.3 (1.6–27) ^e^	Y (17/27)	Y		✓	✓	✓
Muenzer 2017 [30]	P, O, MC, HOS	Multiple countries	Effectiveness	56.3 (18.2–97.6) months ^f^	639	3.1 (1.0, 6.7) ^f^	1.5 (0.3, 4.0) ^f^	6.2 (2.1, 18.2) ^f^	Y (385/626)	NR	Unclear	✓	✓	✓
Żuber 2014 [31]	P, O, SC	Poland	Height outcomes	52–288 weeks	13	3 (<0.1–4.0) ^e^	NR	4.0 (0.3–6) ^e^	Y (12/13)	Y	✓	✓	✓	
Bik-Multanowski 2017 [32]	P, O	Poland	Effectiveness	<0.5–6.0 y	45	NR	NR	Range (0.3–32.5)	NR	NR	✓		✓	✓
Parini 2015 [33]	P, O, SC	Italy	Effectiveness	7.8 (3.0–10.3) years ^e^	17	4.0 (2.0–4.6) ^e^	0.9 (0–3) ^e^	9 (2.3–25.5) ^e^	Y (11/16)	Y		✓	✓	✓
**Retrospective studies**														
Burton 2011 [34]	R, O, MC, HOS	Multiple countries	IRRs	≥12 months	104	NR	NR	8.7 (1.6–39.3) ^e^	Y (52/102)	NR		✓	✓	✓
Pano 2015 [21]	R, OL, MC	Multiple countries	Immunology assessment	12 months	28	3.5 (0.2–6.5) ^e^	NR	4.0 (1.3, 7.5) ^b^	N	Y	✓	✓	✓	
Burton 2010 [35]	R, O, MC, HOS	Multiple countries	Feasibility of home infusions	NR (only duration of home therapy provided)	92	NR	NR	8.5 (3.4, 17.9) ^f^	Y (34/92)	NR		✓	✓	Unclear
Muenzer 2011 [36]	R, O, MC, HOS	Multiple countries	Safety and Effectiveness	22.9 (14.6) months ^c^	124	2.4 (0.3, 4.0) ^f^	1.0 (0.1, 3.0) ^f^	3.6 (1.6) ^c^	Y (61/118 <6 y; 177/278 ≥6 y)	Y	✓	✓	✓	
Racoma 2021 [37]	R, O, SC	The Philippines	Effectiveness	21.1 (10–32) months ^b^	8	7.0 (4.2) ^c^	NR	14.0 (4.0–21.5) ^b^	Y (all: 32/40; treated: 4/8)	NR		✓	✓	✓
Glamuzina 2011 [38]	R, O, SC	UK	Treatment response	22.1 (5–35) months ^b^	11	NR	NR	Range (<1.0–8.7)	Y (7/11)	NR	✓	✓	✓	
Barbier 2013 [20]	R, RCT/OLE, MC	Multiple countries	Immuno-genicity, safety and efficacy	105 weeks	63	NR	NR	Range (5–31)	N	Y			✓	✓
Schulze-Frenking 2011 [39]	R, O, MC	Multiple countries	Growth outcomes	3 years	18	NR	NR	Range (6–19)	N	NR			✓	✓
Lampe 2014 [40]	R, O, MC	Multiple countries	Effectiveness	4.7 (2.0–6.0) years ^a,b^	22	2.8 (0.2–5.0) ^b^	NR	6.8 (1.5–21.0) ^b^	Y (22/22)	NR		✓	✓	✓
Burton 2017 [41]	R, O, MC, HOS	Multiple countries	Survival outcomes	57.8 (10.6–106.2) months ^f^	800	3.3 (1.0, 7.1) ^f^	1.6 (0.3, 4.3) ^f^	6.9 (2.1, 19.8) ^f^	Y (464/800)	NR		✓	✓	✓
do Valle 2020 [42]	R, O, SC	Brazil	Impact of ERT on LVM	5 years	14	3.2 (0.0–6.0) ^e^	NR	6.9 (4.0–12.0) ^e^	Y (14/14)	NR		✓	✓	
Ueda 2020 [43]	R, O, MC	Japan	Safety and effectiveness	336–349 weeks	145	4.0 (0.0, 53.0) ^f^	1.0 (0.0, 12.0) ^f^	10.0 (0.0–54.0) ^f^	Y (72/141)	NR	✓	✓	✓	✓
Tomita 2021 [44]	R, O, SC	Japan	Effectiveness	9.5 (4.5) years ^c^	20	NR	NR	Median 7.0 (IQR: 3.0–12.0)	Y (20/28)	Y	✓	✓	✓	✓
Broomfield 2020 [45]	R, O, MC	UK	Effectiveness	9.3 (0.2, 17.8) years ^e^	78	2.7 (0.3 –15.8) ^b^	NR	6.6 (<0.1–16.0) ^e^	NR	Y	✓	✓	✓	
Patel 2014 [46]	R, O, SC	Japan	Growth outcomes	NR	26	NR	NR	4.5 (2.4) ^c^	Y (35/44)	NR	✓	✓	✓	Unclear
**Cross-sectional studies**														
Jones 2013 [47]	CS, O, MC, HOS	Multiple countries	Growth outcomes	NR (data in study collected for treatment ≤24 months)	133	4.0 (1.5, 8.3) ^f^	2.0 (0.3, 4.8) ^f^	11.3 (2.2 ^c^	Y (66/133)	Y			✓	
Jacques 2016 [48]	CS, O, SC	Brazil	Effect on oxidative stress and cytokines	5.2 (1.5–7.0) years ^b^	8	NR	NR	11.9 (1.5–23.1) ^b^	NR	NR		✓	✓	✓

^a^ Patients treated with idursulfase. ^b^ Mean (range). ^c^ Mean (SD). ^d^ Treated at recommended clinical dosage of 0.5 mg/kg once weekly; other dosages were tested in this study. ^e^ Median (range). ^f^ Median (10th, 90th percentiles). CS—cross-sectional design; ERT—enzyme replacement therapy; HOS—Hunter Outcome Survey; IQR—interquartile range; IRR—infusion-related reaction; IV—intravenous; JROM— joint range of motion; LVM—left ventricular mass; MC—multicenter; MPS II—mucopolysaccharidosis type II; N—no; neuro—neuronopathic; non-neuro—non-neuronopathic; NR—not reported; O—observational design; OL—open-label clinical trial; OLE—open-label extension; P—prospective design; Pbo—placebo; PK/PD—pharmacokinetics/pharmacodynamics; R—retrospective design; RCT—randomized controlled trial; SC—single-center; SD—standard deviation; Tx—treatment; uGAG—urinary glycosaminoglycan; Y—yes.

**Table 3:** Impact of IV idursulfase on clinical outcomes by treatment duration.

Outcome	Treatment Duration	Results Overview
uGAG	4–6 months	Significant reduction in uGAG levels from baseline (diagnosis) vs. nontreated controls after 6 months of treatment (values not disclosed; *p* < 0.01) [24]. Significant reductions in uGAG levels at 6 months with IV idursulfase at 0.15 mg/kg EOW (41%; *n* = 4), 0.5 mg/kg EOW (44%; *n* = 4), and 1.5 mg/kg EOW (58%; *n* = 4) (*p* < 0.0001 for pooled data compared with baseline) in children and adults [13]. Decreases in uGAG levels appeared as early as week 18 (–49.2% change) across all age groups in children [12]. Significant reduction in uGAG levels from baseline to 4 months (362.0 μg/mg creatinine at baseline to 156.0 μg/mg creatinine at 4 months, *p* < 0.001) [11].
	1 year	Significant reductions in uGAG levels at 12 months with IV idursulfase at 0.15 mg/kg EOW (47%; *n* = 4), 0.5 mg/kg EOW (43%; *n* = 4), and 1.5 mg/kg EOW (58%; *n* = 4) (*p* < 0.0001 for pooled data compared with baseline) in children and adults [13]. Reduction of 52.5% in uGAG levels by week 53 with idursulfase weekly; significantly different to placebo (*p* < 0.0001) and idursulfase EOW (*p* = 0.0394); 26/64 patients with either regimen had normalized uGAG levels by week 53 [19]. Significant reduction in uGAG levels in nine adults by 12 months (79.9%, *p* = 0.004); age at treatment start: 30.1 years (range: 21.1–53.9 years) [22]. Decrease in uGAG levels by week 53 (−54.4% change) across all age groups in children. No patients normalized after 1 year of treatment [12]. Significant reduction in uGAG levels from baseline reported between 8 weeks and 52 weeks (*p* = 0.015) [38]. Significant reduction in uGAG levels after 1 year of treatment in all patients (*n* = 17; *p* = 0.0005) [33].
	2 years	uGAG level reduction was maintained between 52 weeks and 120 weeks (*p* = 0.096) after an initial significant reduction at 52 weeks (*p* = 0.015) [38]. Significant reduction in uGAG levels in all patients (*n* = 40; <6-year-old subgroup); in those with elevated uGAG levels (>200 µg/mg creatinine) at baseline (*n* = 34; <6-year-old subgroup), mean (SD) uGAG level in µg/mg creatinine reduced from 592 (188) to 218 (115) after ≥6 months of treatment (*p* < 0.0001) over a mean treatment duration of 22.9 months [36].
	3–3.5 years	Reduction in uGAG levels from baseline after 3 years of treatment (362.0 μg/mg creatinine at baseline to 81.7 μg/mg creatinine at 3 years). Only 31/94 patients had uGAG levels above ULN (127 μg/mg creatinine) by 3 years [11,19]. Reduction in uGAG levels from baseline to 3 years of 70.5% (*n* = 121; significance not reported) [30]. Significant uGAG level reduction of 74.6% in 13 patients ≤5 years of age (*p* = 0.0002) after 3.5 years of treatment [29].
	5 years	After a mean duration of 4.7 years, uGAG levels were reduced by 22–97% vs. baseline in 20/22 patients with neuronopathic MPS II. uGAG levels were increased in two patients (*n* = 1 ADA positive; *n* = 1 ADA negative); both patients experienced four or more somatic improvements despite increased uGAG levels [40]. Initial significant reduction in uGAG levels after 1 year was maintained at 5 years (*n* = 16, *p* = 0.0001) [33].
	7 years	Initial significant reduction in uGAG levels after 1 year was maintained at 7 years (*n* = 12, *p* = 0.002) [33].
	8 years	After 8 years of treatment, significant uGAG level reduction of 162.88 mg/g creatinine (95% CI: 186.68, 139.09 mg/g creatinine) in 39 patients <15 years of age with neuronopathic MPS II, and 147.34 mg/g creatinine (95% CI: 184.26, 110.42 mg/g creatinine) in 21 patients <15 years of age with non-neuronopathic MPS II [43].
	9.5–10 years	uGAG levels reduced from baseline after 9.5 years of treatment (mean at baseline [SD]: 226.5 [109.3] mg/g creatinine to 36.6 [29.1] mg/g creatinine at 9.5 years; significance not reported) [44]. A 10-year pediatric-onset study showed a reduction in uGAG levels ranging from 53.2% to 80.8% across three laboratories and 72 children (patient age not specified). Seven patients had increased uGAG levels (no ERT, *n* = 2; persistent high ADA titers, *n* = 4; ADA titers not available for one patient) [45].
Cardiac	1 year	LVMI was numerically reduced after 12 months of ERT in six patients (>5 years of age) with baseline LVH; two patients reached normal limits. Improvements in other cardiac parameters, including aortic morphology and mitral and tricuspid valve function, were variable, and no clear treatment effect was observed (pooled results for IV idursulfase 0.15, 0.5, and 1.5 mg/kg EOW) [13]. LVMI and LVEF were numerically reduced by 12.4% and 2.8% after 12 months in adult patients with LVH at baseline (n = 6–10; *p* = NS) [22]. LVMI was maintained after 1 year of treatment (70.9–70.4 g/m^2^) in children aged 4–12 years (*n* = 14) [42].
	2–3.5 years	After 2 years of treatment, improvement in LVMI was reported in 3/3 treated patients (4–22 years of age): change from 92 g/m^2^ at baseline to 83 g/m^2^. After 2 years, 2/3 patients had valvular involvement (vs. 3/3 at baseline) and no patients had cardiac hypertrophy [37]. LVMI was reduced from 70.9 g/m^2^ at baseline to 49.9 g/m^2^ after 2 years of treatment and maintained at 3 years (47.2 g/m^2^) in children aged 4–12 years (significance only reported for 5-year reduction) [42]. In six treated patients, there was a significant reduction in LVMI (*p* = 0.032) and IVSd (*p* = 0.05) after 2–3 years of treatment [28]. In HOS patients, LVMI was reduced by 9.3% after 3 years of treatment. In patients without LVH, LVMI did not increase. For patients with LVH at baseline, there was a general trend toward decreased LVMI with treatment, although patient numbers were limited [30]. After 3.5 years of treatment, improvement or stabilization of valve regurgitation (particularly mitral and tricuspid valves) was observed in most ERT-treated patients [29].
	5 years	In children aged 4–12 years, LVMI was significantly reduced over 5 years (*p* = 0.003) [42].
	5–8 years	After 5–8 years of treatment, 12/13 patients showed significant reduction of LVMI compared with baseline. LVMI was particularly improved in 4/13 patients who had overt hypertrophy at baseline (from 136.9–193.6 g/m^2^ to 76.3–131.1 g/m^2^). Severity of valve disease had worsened in four patients and was unchanged in the others [33].
	11–14 years	LVH had resolved in all 6 of 46 pediatric-onset patients who had a degree of LVH at baseline (range of follow-up: 14 months–13.8 years). However, valvular disease was noted to have progressed in 18/46 (39.1%), with 9/46 (19.6%) showing progression of their mitral valve disease, whereas 13/46 (28.3%) had progression of the aortic valvular disease; the length of follow-up was not significantly different in patients whose mitral valve disease progressed (12.7 years) compared with those without progression of valve disease (11.3 years; except for 1 patient with mitral valve disease progression) [45].
Respiratory	1 year	After 12 months, a small statistically non-significant improvement in FVC from baseline was reported in 9/12 patients >5 years of age (from 1.03 L at baseline to 1.10 L at 12 months; *p* = 0.08), whereas FEV_1_ was not improved (pooled results for IV idursulfase 0.15, 0.5, and 1.5 mg/kg EOW) [13]. FVC (percentage predicted) improved (but not statistically significantly), whereas absolute FVC was statistically significantly improved (a change from baseline to week 53 of 0.22 [*p* = 0.0011]) [19]. Clinically meaningful (but not statistically significant) improvements in predicted FVC were reported in adults after 12 months of treatment [22].
	2 years	A trend toward reduction in percentage predicted FVC was seen over a 2-year observation period in 2/4 patients for whom there were available lung data (5–9 years of age) [38].
	3 years	Absolute FVC demonstrated sustained improvements over 3 years of treatment, with minimal changes in percentage predicted FVC. Patients <12 years of age and patients aged 12–18 years demonstrated substantial increases in absolute FVC, with absolute FVC values of (mean [SE]) 0.39 (0.09) L and 0.45 (0.11) L at 3 years, respectively. In patients >18 years of age, a decrease of 0.04 L was seen at 36 months [11]. Based on HOS data, absolute FVC increased each year of treatment compared with baseline up to 3 years. After 3 years, median (10th, 90th percentiles) change from baseline was 0.3 (−0.2, 1.1) L (*n* = 23). After 3 years, median increase from baseline in absolute FEV_1_ was 0.2 (−0.3, 0.9) L (*n* = 22) [30].
	8 years	Small changes in FVC (median [95% CI]: 0.060 [−0.088, 0.603]) and FEV_1_ (median [95% CI]: 0.040 [−0.091, 0.514]) over 8 years of treatment suggested stable respiratory function with long-term ERT [43].
	10 years	After ~10 years of treatment in a pediatric-onset population, absolute FVC increased; however, predicted FVC decreased, particularly in the second decade. Significantly better lung function was reported in patients who started IV idursulfase at a young age (<8 years of age; *n* = 11; percentage predicted FVC was 69% [range: 34–86%]) than in those who started at a later age (>8 years of age; *n* = 13; predicted percentage FVC was 48% [range: 25–108%]) (*p* = 0.045) [45].
Hepatosplenomegaly	3–6 months	Based on HOS data, significant reductions in liver volume after 6 months of IV idursulfase treatment were reported in both age subgroups of patients with an enlarged liver at baseline (<6 years and ≥6 years) [36]. Significant reductions were seen in liver and spleen volumes at 4 months, which were maintained after 3 years of treatment [11]. Significant reductions were seen in liver and spleen volumes after 6 months (*p* < 0.0001 and *p* = 0.0043, respectively; pooled results for IV idursulfase 0.15, 0.5, and 1.5 mg/kg EOW) [13]. Liver and spleen volumes decreased by >20% after 18 weeks of treatment. About 80% of patients with hepatomegaly at baseline (n = 20/25) had normal liver volume after 18 weeks of treatment [19]. In a pediatric population, ~20% reductions in liver and spleen volumes were reported as early as week 18 (significance not reported) [12]. In adults, most reductions in liver and spleen volumes were observed in the first 3 months of treatment [22].
	1 year	Significant reductions were seen in liver and spleen volumes after 12 months (*p* < 0.0001). Liver volume was reduced to within normal limits in 6/9 patients with hepatomegaly at baseline and remained within normal limits in the other 3 patients. After 12 months, all patients had a spleen volume within normal limits (pooled results for IV idursulfase 0.15, 0.5, and 1.5 mg/kg EOW) [13]. Liver and spleen sizes were significantly reduced (~25% reduction) compared with placebo after 12 months of treatment (*n* = 32 [age range: 6–26 years]). About 80% of patients with hepatomegaly at baseline had normal liver volume after 53 weeks of treatment. Most patients had normal spleen volume at baseline [19]. In a pediatric population, liver and spleen volumes were reduced by 17.4% and 20.6%, respectively, by 53 weeks compared with baseline (significance not reported); all patients had an enlarged liver or spleen at baseline [12]. In adults, significant reductions in liver volume (33.2%) and spleen volume (31.0%) were reported after 12 months of treatment (*p* = 0.002) [22].
	3–6 years	Significant reductions in liver and spleen volumes were maintained after 3 years of treatment [11]. Based on HOS data, after 3 years, 62.4% of patients no longer had a palpable liver and 78.7% of patients no longer had a palpable spleen between baseline and 3 years after treatment initiation; median (10th, 90th percentiles) palpable liver size changed from 6.0 (3.0, 11.0) cm to 3.0 (1.0, 6.0) cm, and median (10th, 90th percentiles) palpable spleen size changed from 5.0 (1.5, 9.0) cm to 2.0 (1.0, 7.8) cm [30]. After 3–6 years of treatment, of patients who were still receiving ERT at the time of the study and patients who had discontinued treatment, respectively, 11 and 23 experienced no change in liver size, whereas 2 and 3 experienced an improvement. The two patients who were still receiving ERT and experienced an improvement started treatment at 12.5 years of age (6 years of treatment) and 5 years of age (2 years of treatment). The three patients who had discontinued treatment and experienced an improvement started treatment at 6 years of age (2.5 years of treatment), 9.5 years of age (3.4 years of treatment), and 14 years of age (2 years of treatment) [32]. All 22 patients with severe disease (1.5–21 years of age) experienced reductions in liver size and/or spleen size after 4.7 years of ERT [40]. Significant amelioration of hepatomegaly over 3.5 years of treatment in patients aged >5 years to ≤12 years; this was not reported for patients ≤5 years or >12 years of age. Splenomegaly was not significantly improved in any of these age groups [29].
	5–8 years	Hepatosplenomegaly was significantly “slightly improved or better” at the end of a 6.5-year evaluation period; 46.0% of patients <15 years of age (40/87) and 38.0% of patients ≥15 years of age (19/50) showed improvement in hepatosplenomegaly [43]. In patients >2.3 years of age, organomegaly decreased progressively and steadily over time. Differences from baseline were significant for spleen size (at 5–6 years [*n* = 15], *p* = 0.0005; at 7–8 years [*n* = 12], *p* = 0.004) and liver size (at 5–6 years [*n* = 16], *p* < 0.0001; at 7–8 years [*n* = 12], *p* = 0.0005) [33].
6MWT	4–6 months	Significant improvement in 6MWT outcome results were reported after 4 months of treatment (increase in walking distance of 14 m, *p* < 0.001) [11]. No substantial changes in the mean 6MWT distance were seen in any treatment group during the 6-month double-blind phase based on pooled results for IV idursulfase 0.15, 0.5, and 1.5 mg/kg EOW dosages [13].
	1 year	After 1 year of treatment, walking distance in the 6MWT increased by 10.9% with IV idursulfase 0.5 mg/kg EOW and by 27.9% with IV idursulfase 1.5 mg/kg EOW (significant improvement from 398 m to 445 m [*p* = 0.013] for pooled study dosages [IV idursulfase 0.15, 0.5, and 1.5 mg/kg EOW]) [13]. Walking distance in the 6MWT increased after 1 year of treatment by 44.3 m vs. 7.3 m with placebo (*p* = 0.0131) (*n* = 32) [19]. In adults, in the 6MWT, walking distance increased by 54.5 m (37.4% change, *p* = 0.109) (*n* = 7/10) after 1 year of treatment. Four patients showed clinically meaningful improvement (increase of ≥54 m), whereas one patient with normal 6MWT walking distance at baseline showed a decline of 71 m [22].
	2 years	Only 2/27 patients had complete 6MWT data for the 2-year period (one decline [of 91.4 m] and one improvement [of 172 m]) [37]. 6MWT distance was measured in 4 of 11 patients (5–9 years of age, 1 with neuronopathic MPS II). The range of the increase in 6MWT results over 2 years of treatment (*n* = 4) was 35–97 m. The other patients were not able to complete the test because they were too young or were noncompliant due to CNS disease [38]. Significant improvement in 6MWT results were reported after 20 months of treatment (+42 m) [11].
	3–3.5 years	Significant improvements in 6MWT results were reported at all time points up to 36 months except for one at 30 months. At 36 months, the largest increase was observed in the >18-year-old group (*n* = 24; +48 m), followed by the <12-year-old group (*n* = 41; +8 m) and the 12–18-year-old group (*n* = 29; +0.7 m) [11]. Based on HOS (*n* = 26/639), a median (10th, 90th percentiles) improvement in walking distance in the 6MWT of +41 (−138, +158) m or +10.6% (−33.6%, +50.8%) was observed after 3 years of treatment [30]. After ~3.5 years of treatment, patients ≤5 years of age (*n* = 3) and >12 years of age (*n* = 1) showed ~20% improvement, whereas in patients >5 years and ≤12 years of age (*n* = 2), one improved (+37%) and one declined (−13%). Data were available only for 6/27 patients owing mostly to noncompliance in the youngest patients [29].
	6 years	In 13 patients who were still receiving ERT at the time of the study (3 months–12.5 years of age) with up to 6 years of treatment, an increase in 6MWT distance was reported in 10 patients, no change in 2 patients, and a decrease in 1. Overall, there was a worsening in 6MWT results in 15 patients who had discontinued treatment, no change in 14 patients, and an improvement in 1 patient [32].
	7–8 years	After 7 years of treatment, 6MWT distance walked was stable or improved (mean: +70.8 m [range: 0–174.0 m]) in patients with non-neuronopathic MPS II (*n* = 5; 6.0–16.3 years of age). In patients with neuronopathic MPS II (*n* = 11; 5.2–15.7 years of age), no improvement was observed and almost all patients were poorly testable due to hyperactive behavior or their using a wheelchair for mobility [33]. After 8 years of treatment, in patients with non-neuronopathic MPS II, 6MWT walking distance increased by 89.0 m in those <15 years of age (*n* = 7) and 27.9 m in those ≥15 years of age (*n* = 8). After the same treatment period, in patients with neuronopathic MPS II, 6MWT walking distance decreased by 22.3 m in those <15 years of age (*n* = 10) and increased by 55.0 m in the patient ≥15 years of age (*n* = 1) [43].
	10 years	After ~10 years of treatment in 18 children starting treatment at ~7 years of age, a median improvement of +67 m (range: −129 m to +292 m) or 17.2% (range: −26.0% to +52.0%) was reported [45].
Safety and immunogenicity	1–6 months	In a HOS analysis, nearly all IRRs (65 reported in total) occurred during the first 3 months of therapy in 28/104 patients: 5 patients experienced an IRR after 3 months, and only 2 of these patients experienced an IRR after 6 months [34]. A 3-year analysis showed that the percentage of patients with an IRR peaked by month 3 of treatment (~40%) and declined thereafter [11]. In a 7-year observational study, IRRs were reported as early as 1 month after treatment [33].
	1 year	In clinical studies, after 1 year of treatment, most children and adults experienced at least one AE, although most were mild or moderate in severity [12,13,19]. At least one serious AE was experienced by 28.1–46.4% of treated patients in studies involving children and adults [12,19] and by 20% in a study assessing adults only [22], with the majority of serious AEs considered unrelated to treatment. The most commonly reported AEs included pyrexia, headache, pharyngitis, upper respiratory tract infections, rhinitis, abdominal pain, urticaria, and vomiting [12,19,22]. Based on a 1-year HOS analysis, 65 IRRs, mostly mild to moderate in severity, were reported in 33 (31.7%) of 104 patients, with most patients reporting IRRs before 6 months of treatment [34]. A 3-year analysis showed that the percentage of patients with an IRR dropped from a ~40% peak at 3 months to ~15% after 12 months, with a decline thereafter [11]. Within the first year of treatment in a post-marketing study evaluating a maximum of up to 8 years of treatment, ADR incidence was 46.2% in patients with treatment history and 30.1% in those with no treatment history [43]. Reductions in both liver size and uGAG response after 1 year of treatment appeared to be associated with both neutralizing antibody status and genotype [21]. Antibody development after 1 year of treatment did not affect the reduction in uGAG levels or the changes in liver and spleen volumes, 6MWT results, or percentage FVC [13].
	2–3 years	Over 3 years of treatment, 59.6% of patients experienced at least one treatment-related AE and 28.7% experienced at least one serious AE. The percentage of patients with an IRR was substantially less after 2–3 years of treatment than after the peak at 3 months (<5% vs. ~40%) [11]. Over ~3 years of treatment (from HOS data), 74.7% of patients reported AEs, with 14.3% and 11.9% of these probably related to treatment and possibly related to treatment, respectively. These AEs were mostly mild or moderate in severity. In total, 34.8% of patients (81/233) reported at least one IRR. The most commonly reported AEs were those classified as infections and infestations, followed by those classified as respiratory, thoracic, and mediastinal disorders [30].
	5–7 years	Over a 7-year observational treatment period, IRRs were reported in 23.5% of patients (4/17). The most common IRRs reported over the 7-year period were itching and urticaria [33]. In patients with neuronopathic MPS II, 18.0% of patients (4/22) experienced at least one IRR at any time over the course of treatment (all four occurred over 5–6 years of treatment). No other AEs were observed [40]. In an 8-year post-marketing study, ADR incidence slightly increased from 46.2% at 1 year of treatment to 55.1% after 5 years of treatment in patients with treatment history and from 30.1% after 1 year of treatment to 51.8% after 5 years of study in those with no treatment history [43].
	8 years	Over up to a maximum of 8 years of treatment in a post-marketing study, serious AEs were reported in 42.8% of patients (62/145); the most common class of serious AEs was respiratory, thoracic, and mediastinal disorders [43]. No significant correlation with anti-idursulfase antibodies was observed in ADR incidence, hypersensitivity-related ADRs, or IRRs [43].

6MWT—6-min walk test; ADA—anti-drug antibody; ADR—adverse drug reaction; AE—adverse event; CI—confidence interval; CNS—central nervous system; EOW—every other week; ERT—enzyme replacement therapy; FEV_1_—forced expiratory volume in the first second; FVC—forced vital capacity; HOS—Hunter Outcome Survey; IRR—infusion-related reaction; IV—intravenous; IVSd—interventricular septum thickness during diastole; LVEF—left ventricular ejection fraction; LVH—left ventricular hypertrophy; LVMI—left ventricular myocardial index; MPS II—mucopolysaccharidosis type II; NS—not significant; SD—standard deviation; SE—standard error of the mean; uGAG—urinary glycosaminoglycan; ULN—upper limit of normal.

**Table 4:** Impact of IV idursulfase on clinical outcomes in patients who started treatment when younger than 18 months.

Outcome	Results Overview
uGAG	A reduction in uGAG levels occurred across all age groups after 53 weeks of treatment; treatment effect appeared as early as week 18 (−49.2% change) and was maintained to week 53 (−54.4% change). No patients normalized by EOS. Of the 28 patients in the study, 4 were <2 years of age [12]. A significant uGAG level reduction from baseline was reported from 8 weeks to 52 weeks of treatment (*p* = 0.015), with no significant change between 52 weeks and 120 weeks (*p* = 0.096). This study included 3/11 patients <18 months of age [38]. A significant reduction in uGAG levels was reported in all patients (*n* = 40) and in those with elevated baseline uGAG levels (>200 µg/mg creatinine) who were <6 years of age and had received ≥6 months of treatment (*n* = 34); mean (SD) uGAG level (µg/mg creatinine) was reduced from 592 (188) at baseline to 218 (115) after treatment (*p* < 0.0001). Of the 124 patients in the study, 11 were <12 months of age [36]. Over a maximum of up to 8 years of treatment in a post-marketing study, significant reductions in uGAG levels in patients <15 years of age were reported: mean (95% CI) reductions were 162.88 (186.68, 139.09) mg/g creatinine in 39 patients with severe MPS II and 147.34 (184.26, 110.42) mg/g creatinine in 21 patients with mild MPS II. The median (range) age in the <15-year-old subgroup was 6 (0–14) years [43]. After 10 years of treatment, a pediatric study showed a reduction in uGAG levels ranging from 53.2% to 80.8% across three laboratories and 72 children (patient age not specified). Seven patients had increased uGAG levels (no ERT, *n* = 2; persistent high ADA titers, *n* = 4; ADA titers not available for one patient) [45].
Cardiac	After ~3 years of follow-up in a single patient (ERT started at 1 year of age; disease diagnosed prenatally), improvements in IVSd and LVMI were reported (change in IVSd from baseline to end of ERT: 2.88 to −0.56; change in LVMI: 0.10 to −0.03) [28]. Mitral valve disease progressed after ~10 years of treatment in 35/58 children (60%) who started treatment at a median (range) age of 3 years (10 months–9.2 years). LVH resolved after ERT in all six patients who presented with LVH at baseline (range of follow-up: 14 months–13.8 years). The number of patients <18 months of age in this study is unclear [45].
Lung	A post-marketing study evaluating ERT up to a maximum of 8 years of treatment reported lung outcomes in patients in the age range 0–14 years (median age, 6 years); therefore, the impact on patients <18 months was unclear [43]. Glamuzina et al. remarked that the three patients <18 months of age included in their study (10, 13, and 14 months) were too young to undergo lung function tests [38].
Hepatosplenomegaly	After 3 years of ERT in a pediatric population, liver volume and spleen volume were reduced by 17.4% and 20.6%, respectively, by 53 weeks vs. baseline, with 20–40% reductions reported as early as week 18 (statistical significance not reported); all patients had an enlarged liver and/or spleen at baseline. Of the 28 patients in the study, 4 were <2 years of age [12]. Hepatosplenomegaly was significantly “slightly improved or better” at the end of a 6.5-year evaluation period; 46.0% of patients <15 years of age (40/87) showed improvement in hepatosplenomegaly. The median (range) age in the <15-year-old subgroup was 6 (0–14) years [43].
6MWT	Glamuzina et al. remarked that the three patients <18 months of age included in their study (10, 13, and 14 months) were too young to undergo 6MWT [38]. Over up to a maximum of 8 years of treatment in a post-marketing study, for patients <15 years of age, change in 6MWT walking distance was +89 m in those with non-neuronopathic MPS II (*n* = 7) and −22.3 m in those with neuronopathic MPS II (*n* = 10). The median (range) age in the <15-year-old subgroup was 6 (0–14) years [43].
Safety and immunogenicity	The majority of children (1.4–7.5 years of age) exposed to 1 year of treatment experienced at least one mild or moderate AE, with 46.4% experiencing at least one serious AE. Of the 28 patients in the study, 4 were <2 years of age [12]. After ~2 years of treatment (from HOS data), 69 nonserious IRRs were reported in 33 patients <6 years of age (26.6%); 28 serious AEs were reported in 16 patients (12.9%). Of the 124 patients in the study, 11 were <12 months of age [36]. Over up to a maximum of 8 years of treatment in a post-marketing study, 53.9% of patients <15 years of age experienced an ADR (*n* = 48/89); of the ADRs reported, 23.6% were attributed to general disorders and administration-site conditions. The median (range) age in the <15-year-old subgroup was 6 (0–14) years [43].

6MWT—6-min walk test; ADA—anti-drug antibodies; ADR—adverse drug reaction; AE—adverse event; CI—confidence interval; EOS—end of study; ERT—enzyme replacement therapy; HOS—Hunter Outcome Survey; IRR—infusion-related reaction; IV—intravenous; IVSd—interventricular septum thickness during diastole; LVH—left ventricular hypertrophy; LVMI—left ventricular myocardial index; MPS II—mucopolysaccharidosis type II; SD—standard deviation; uGAG—urinary glycosaminoglycan.

## Data Availability

The data included in this report are from the published literature; all articles meeting the search criteria are listed and full publication details are provided.

## Supplementary Materials

The following supporting information can be downloaded at: https://www.mdpi.com/article/10.3390/ijms25168573/s1.
