# Supplementary material for: Intravenous Idursulfase for the Treatment of Mucopolysaccharidosis Type II: A Systematic Literature Review
Source: Int J Mol Sci. 2024 Aug 6;25(16):8573. doi: 10.3390/ijms25168573 (PMC11354461; doi:10.3390/ijms25168573)
Supplement: Supplementary file 1 [file ijms-25-08573-s001.zip › ijms-3104642-supplementary.pdf]

Supplementary Material

Table S1. Search strings for the systematic literature review.

Embase

Search no.	Search	Result	Type
1	(((mucopolysaccharidosis or MPS) adj (two or type 2 or II)) or (hunter* adj (syndrome or disease))).tw.	2231	MPS II
2	exp Hunter syndrome/dm, dt, th [Disease Management, Drug Therapy, Therapy]	584	
3	(treatment* or therap* or surg* or (gen* adj therap*) or (enzyme adj replacement) or ERT or Iduronate sulfatase).tw.	11 426 474	Treatments
4	exp cell penetrating peptide/ or cargo technology.tw. or exp drug delivery system/	415 343	
5	(1 or 2) and (3 or 4)	1501	
6	case report/	2 837 507	Case reports from 2017 onward
7	(case study or case report or (case adj3 present*) or case series or clinical course).tw.	1 048 765	
8	6 or 7	3 191 518	
9	limit 8 to yr="2017 -Current"	874 125	
10	5 and 9	160	Boolean combinations
11	5 or 10	1501	
12	limit 11 to English language	1423	
13	limit 12 to (books or chapter or conference abstract or "conference review" or editorial or erratum or letter or note or "review" or short survey or tombstone)	810	
14	12 not 13	613	
15	limit 14 to (animals and animal studies)	51	
16	14 not 15	562	
17	limit 16 to human	521	

MEDLINE

Search no.	Search	Result	Type
1	(((mucopolysaccharidosis or MPS) adj (two or type 2 or II)) or (hunter* adj (syndrome or disease))).tw.	1365	MPS II
2	exp Hunter syndrome/dm, dt, th [Disease Management, Drug Therapy, Therapy]	274	
3	(treatment* or therap* or surg* or (gen* adj therap*) or (enzyme adj replacement) or ERT or Iduronate sulfatase).tw.	8 412 449	Treatments
4	exp cell penetrating peptide/ or cargo technology.tw. or exp drug delivery system/	169 338	
5	(1 or 2) and (3 or 4)	668	
6	case report/	2 313 692	Case reports from 2017 onward
7	(case study or case report or (case adj3 present*) or case series or clinical course).tw.	772 662	

8	6 or 7	2 602 815	
9	limit 8 to yr="2017 -Current"	528 187	
10	5 and 9	76	Boolean combinations
11	5 or 10	668	
12	limit 11 to English language	634	
13	limit 12 to (books or chapter or conference abstract or "conference review" or editorial or erratum or letter or note or "review" or short survey or tombstone)	99	
14	12 not 13	535	
15	limit 14 to (animals and animal studies)	76	
16	14 not 15	459	
17	limit 16 to human	347	

Cochrane Library

Search no.	Search	Result	Type
1	(((mucopolysaccharidosis or MPS) adj (two or type 2 or II)) or (hunter* adj (syndrome or disease))).tw.	81	MPS II
2	exp Hunter syndrome/dm, dt, th [Disease Management, Drug Therapy, Therapy]	3	
3	(treatment* or therap* or surg* or (gen* adj therap*) or (enzyme adj replacement) or ERT or Iduronate sulfatase).tw.	1 149 192	Treatments
4	exp cell penetrating peptide/ or cargo technology.tw. or exp drug delivery system/	8273	
5	(1 or 2) and (3 or 4)	69	
6	case report/	4	Case reports from 2017 onward
7	(case study or case report or (case adj3 present*) or case series or clinical course).tw.	14 590	
8	6 or 7	14 593	
9	limit 8 to yr="2017 -Current"	6129	
10	5 and 9	0	Boolean combinations
11	5 or 10	69	
12	limit 11 to English language	65	
13	limit 12 to (books or chapter or conference abstract or "conference review" or editorial or erratum or letter or note or "review" or short survey or tombstone)	4	
14	12 not 13	61	

*(asterisk) was used to search for multiple variants of a word (i.e., singular/plural/conjugations etc.) all at once.
ERT—enzyme replacement therapy; MPS—mucopolysaccharidosis.
